# Functional cervicothoracic boundary modified by anatomical shifts in the neck of giraffes

**DOI:** 10.1098/rsos.150604

**Published:** 2016-02-03

**Authors:** Megu Gunji, Hideki Endo

**Affiliations:** 1 The University Museum, The University of Tokyo, 7-3-1 Hongo, Bunkyo-ku, Tokyo 113-0033, Japan; 2 Department of Global Agricultural Sciences, Graduate School of Agricultural and Life Sciences, The University of Tokyo, 1-1-1 Yayoi, Bunkyo-ku, Tokyo 113-8657, Japan

**Keywords:** axial skeleton, cervicothoracic boundary, giraffe, homeotic transformation, musculoskeletal structure, neck elongation

## Abstract

Here we examined the kinematic function of the morpho- logically unique first thoracic vertebra in giraffes. The first thoracic vertebra of the giraffe displayed similar shape to the seventh cervical vertebra in general ruminants. The flexion experiment using giraffe carcasses demonstrated that the first thoracic vertebra exhibited a higher dorsoventral mobility than other thoracic vertebrae. Despite the presence of costovertebral joints, restriction in the intervertebral movement imposed by ribs is minimized around the first thoracic vertebra by subtle changes of the articular system between the vertebra and ribs. The attachment area of *musculus longus colli*, mainly responsible for ventral flexion of the neck, is partly shifted posteriorly in the giraffe so that the force generated by muscles is exerted on the cervical vertebrae and on the first thoracic vertebra. These anatomical modifications allow the first thoracic vertebra to adopt the kinematic function of a cervical vertebra in giraffes. The novel movable articulation in the thorax functions as a fulcrum of neck movement and results in a large displacement of reachable space in the cranial end of the neck. The unique first thoracic vertebra in giraffes provides higher flexibility to the neck and may provide advantages for high browsing and/or male competition behaviours specific to giraffes.

## 1. Introduction

The mammalian vertebral column comprises successive units organized into five series that are highly conserved and easily recognized: cervical, thoracic, lumbar, sacral and caudal. Cervical vertebrae are traditionally distinguished from thoracic vertebrae by the absence of movable rib articulations. The number of cervical vertebrae has remained constant at seven for at least 200 Myr, despite variable counts in other regions of the mammalian vertebral column and in the cervical vertebrae of other vertebrate classes [1–3]. The diversity of neck length and shape in mammals has evolved under the rigorous constraint of cervical number [3]. The long neck of giraffes also follows the cervical constraint; therefore, each cervical vertebra is prominently longer than that of the common short-necked ruminants, including okapi [4,5].

A previous study raised the possibility that giraffes had gained an additional rib-bearing cervical vertebra in association with their neck elongation [6]. Skeletal comparisons between giraffe and okapi revealed morphological transformations of the vertebrae between the seventh cervical vertebra (C7) and second thoracic vertebra (T2) in giraffes. The most notable transformation is observed at C7. In okapi, C7 is obviously differentiated from other cervical vertebrae by its exhibition of an elongated neural spine and lack of the transverse foramen and ventral tubercles. Conversely, C7 in the giraffe presents the characteristics that are generally observed between C3 and C6: it contains the transverse foramen, ventral tubercles, short neural spine and vertebral body elongation [6–8]. In the case of giraffes, various morphological features generally observed in C7 are found in T1. For example, the vertebral length of T1 of the giraffe represents an intermediate value between the cervical and other thoracic vertebrae [4,6]. The facet type of vertebral articulation is transitional from the radial type to the tangential type at the joint between T1 and T2 in the giraffe, whereas the transitional point of articulation lies between C7 and T1 in okapi, which represents the typical ruminant pattern [6,7]. Additionally, the configuration of roots of the brachial plexus supports the structural similarity between T1 of the giraffe and C7 of okapi [6]. From the osteological and neurological evidences, previous studies have proposed the idea that T1 of giraffe is homologous with C7 of okapi [6,7].

Despite many morphological similarities between T1 of the giraffe and C7 of okapi, prior studies have criticized the idea of a rib-bearing cervical vertebra in giraffe by the presence of the movable rib in T1: a conclusive criterion for classifying a vertebra as a thoracic vertebra [4,5,9,10]. Additionally, the ribs articulating to T1 connects to the sternum. Therefore, T1 of the giraffe has been regarded as merely one of the thoracic vertebrae, and the functional insight of this transformation of the vertebral shape has not been mentioned. Revealing the specific function of these modified vertebrae will allow us to understand the evolutionary and ethological advantages of the morphological transformations of the vertebrae in giraffe. Given the general linkage between the vertebral shape and mobility in vertebrates [11–24], morphological similarities imply that T1 of the giraffe bears a kinematic resemblance to C7 of okapi. The C6/C7 and C7/T1 joints are highly specialized, facilitating the ventral flexion of the head–neck complex in quadrupedal mammals [25]; hence, we can presume that giraffe may have gained an additional movable joint contributing to the ventral flexion of the neck into the T1/T2 joint. Here we evaluated the ability of the ventral flexion of vertebrae around the cervicothoracic area in giraffe and described the musculoskeletal structure of the neck to reveal a functional significance of the morphological shift of the vertebral column.

## 2. Material and methods

### 2.1 Analyses of sagittal mobility of each vertebra

**Figure 1.**
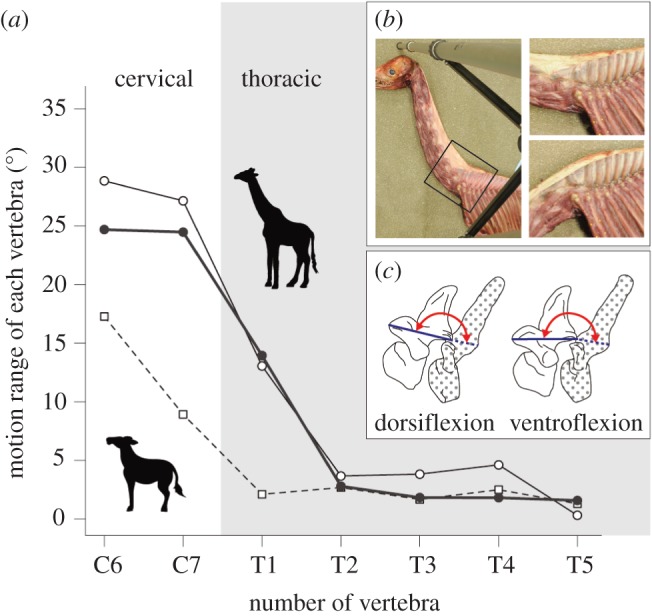
Results and methods of the flexion experiment. (*a*) Estimated motion range of each vertebra from C6 to T5 obtained by the experiment. Open circles indicate the estimated value of the motion range in a giraffe (NSMT-M43180) and filled circles indicate those in the remaining giraffe (NSMT-M43076). Open squares represent the estimated motion range in okapi (KPM-NF1005125). The shaded area indicates the thoracic region. (*b*) The flexion experiment of the neck using the carcasses. The black square in the left indicates the region in which we analysed the vertebral mobility. The upper right shows the posture at the moment of the maximal dorsal flexion of the giraffe neck, and the lower right displays the posture at the moment of the maximal ventral flexion. (*c*) Procedure for estimating the vertebral mobility. The motion range of each vertebra was estimated from the angles between adjacent vertebrae both in maximal dorsal (left) and ventral flexion (right). Angles were measured from the lines connecting the anterior and posterior edge of the articular processes and are shown as arc arrows.

For evaluating the mobility of each vertebral joint, we conducted the flexion experiment using giraffe and okapi carcasses. We forcibly moved their neck from the maximum ventral flexion posture to the maximum upraised posture, and recorded the positional relationship of each vertebra in both postures by using photography and computed tomography. We calculated the angle of a vertebra relative to the posterior adjacent vertebra in the cases of the maximal dorsal and ventral flexion of the neck (figure 1*b*). The angle between two adjacent vertebrae was measured with reference to the line connecting the anterior edge and posterior edge of the articular process in the vertebrae (figure 1*c*). The motion range of each vertebra in a dorsoventral direction was evaluated by the difference between the angle in the dorsal and ventral flexion. In one giraffe and okapi, the angle was calculated from three-dimensional data obtained by computed tomographic imaging. We constructed the three-dimensional model from the computed tomography images by using the Osirix imaging software (v. 6.5.2; http://www.osirix-viewer.com/ContributionOsiriX.html), and then measured the mobility of each vertebral joint. In the other giraffes, we removed some epaxial muscles located on one side to observe the positional relationships of the articular processes, which were then photographed from a lateral view in both postures (figure 1*b*). In this study, we measured the motion range of the two most caudal cervical and first five thoracic vertebrae.

### 2.2 Description of musculoskeletal structure

We described the morphological relationships between the thoracic vertebrae and ribs in giraffe and okapi to reveal the osteological restriction on intervertebral flexion movement imposed by ribs. The costovertebral joints were observed using dried skeletons of giraffe and okapi. For inferring the effect of rib articulation against vertebral movement, we described the positions of costal foveae on thoracic vertebrae and the positional relationships between two adjacent vertebrae and a rib.

We dissected giraffe and okapi carcasses and then described the muscular system generating the force resulting in the ventral flexion of the vertebrae for inferring the active ability of the vertebral flexion of giraffe. Gross anatomical dissections of giraffe and okapi were conducted sequentially from the superficial to the deep layer. Specimens were fresh-frozen and thawed before dissection. We focused on *musculus longus colli*, located on the ventral surface of the vertebral body, that is the main muscle responsible for ventral flexion of the neck [26]. Previous studies about the anatomy of the giraffe have noted that *musculus longus colli* represents a normal structure in giraffes; however, they reported only the structure of the cervical part of the muscle without addressing the muscular structure around the cervicothoracic area displaying the particular morphological transformations of the vertebrae [27,28]. Solounias [6] mentioned differences in the attachment area of the muscle between giraffe and okapi, although the muscular structure was not reported in detail. We described the origins and insertions of the muscle in giraffe and okapi, following which the active mechanism of lowering their head and neck was discussed.

The terminology of the vertebrae and muscles was consistent with the prior works of the vertebral column of Giraffidae [6,8].

### 2.3 Specimens

In the flexion experiment, we used the carcasses of two giraffes and one okapi, with all being former zoo animals. All were newborn specimens of a few days old, possessing the intact axial skeleton because infant specimens allow easier conduct of the flexion experiment. The skeletal structure around the cervicothoracic area was described by observing dried skeletons of eight adult giraffes and five okapis. Data of the muscular structure were obtained from the carcasses of six giraffes and one okapi. Individual information regarding the specimens used in this study is listed in table 1.

**Table 1.** Materials used in this study. (Asterisks mean the specimen used in each work. We conducted the macroscopic dissection in UMUT-13037, 13055, 13056, 15052, and observed the skeletal structure after making the skeletal specimens in these materials. Institutional abbreviations are: KPM, Kanagawa Prefectural Museum of Natural History (Kanagawa, Japan); MNHN, Muséum national d’histoire naturelle (Paris, France); NSMT, National Museum of Nature and Science, Tokyo (Tokyo, Japan); UMUT, The University Museum, The University of Tokyo (Tokyo, Japan).)

species		specimen	age	sex	flexion experiment	skeletal observation	dissection	donor
giraffe	*Giraffa camelopardalis*	UMUT-09147	8-year old	male		*		Kobe Oji Zoo
		UMUT-10071	15-year old	female		*		Hamamatsu Zoological Garden
		UMUT-10072	21-year old	male		*		Hamamatsu Zoological Garden
		UMUT-13037	19-year old	male		*	*	Hirakawa Zoological Park
		UMUT-13055	8-year old	male		*	*	Kobe Oji Zoo
		UMUT-13056	5-year old	male		*	*	Chiba Zoological Park
		UMUT-14140	adult	male		*		no information
		UMUT-15052	11-year old	male		*	*	Toyama Municipal Family Park
		NSMT-M43076	newborn	female	*		*	Tama Zoological Park
		NSMT-M43180	newborn	male	*		*	Tama Zoological Park
okapi	*Okapia johnstoni*	KPM-NF1005125	newborn	male	*		*	Zoorasia Yokohama Zoological Gardens
		NSMT-M-35837	11-year old	female		*		Ueno Zoological Gardens
		MNHN-ZM-AC-1959-262	infant	female		*		no information
		MNHN-ZM-AC-1968-119	adult	female		*		no information
		MNHN-ZM-AC-1975-101	adult	male		*		no information
		MNHN-ZM-AC-1990-43	adult	male		*		no information

## 3. Results

### 3.1 Estimated sagittal mobility of each vertebra

Our flexion experiment revealed that the motion range of each vertebra gradually declined through three successive vertebrae at the cervicothoracic area and that the pattern of decline was different between giraffe and okapi (figure 1*a*). In giraffe, the estimated ranges of motion were clearly large in C6 and C7 and decreased progressively in the caudal direction from C7 to T2. T1 of the giraffe exhibited an intermediate motion range between C7 and T2 and possessed a greater mobility than the remaining thoracic vertebrae (see the electronic supplementary material, movie S1). The motion range of each vertebra maintained a low value between T2 and T5 (figure 1*a*). Conversely, in okapi, the reduction of the motion range was confirmed between C6 and T1. The motion range of C7 showed an intermediate value between C6 and T1, and the ranges between T1 and T5 represented a low mobility (figure 1*a*). Our results showed that the decreasing point of the vertebral mobility was shifted posteriorly in giraffes, and that the intermediate mobility was observed in T1 of the giraffe and C7 of okapi. This indicates the possibility that giraffes have acquired an additional movable articulation into the T1/T2 joint and that the kinematic characteristic of T1 of the giraffe is similar to C7 of okapi in the dorsoventral movement of the neck.

### 3.2 Skeletal structure

All thoracic vertebrae of giraffes and okapi, with exception of T1 of the giraffe, possessed three costal foveae at a lateral surface of each vertebra: cranial costal fovea, caudal costal fovea and costal fovea of the transverse process. The cranial costal fovea was located on the lateral side of the outer border of the cranial convex extremity of the vertebral body, and the caudal costal fovea was positioned on the exterior edge of the caudal concave extremity of the vertebral body. However, the cranial and caudal costal foveae in T1 of the giraffe were not confirmed. T1 possessed an isolated costal fovea located on the central area of the lateral side of the vertebral body below the transverse process.

**Figure 2.**
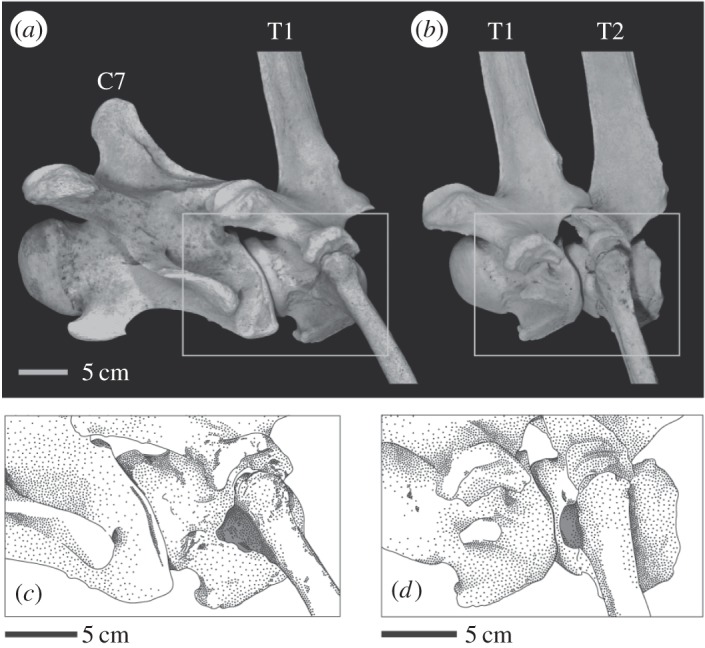
Osteological restriction against vertebral flexion imposed by rib articulation. The first rib of the giraffe attaches on the central area of the lateral surface of T1. The capitulum of the first rib does not connect to the caudal edge of the vertebral body of C7 (*a*,*c*). The second rib of the giraffe articulates with the cranial part of T2. The capitulum of the rib does not disturb the joint between T1 and T2 (*b*,*d*). The shaded part of the ribs in (*c*,*d*) indicates the position of the capitulum of the rib. The skeletal specimen is UMUT-14140.

According to our skeletal observations, the capitulum of the first rib in giraffe made contact with the isolated fovea and did not touch the caudal part of the vertebral body of C7 (figure 2*a*,*c*). The second rib articulated with T2 at the cranial costal fovea and costal fovea of the transverse process, without contacting the vertebral body of T1 (figure 2*b*,*d*). In the third rib, the capitulum of the rib was jointed to the socket formed by the caudal costal fovea of T2 and the cranial costal fovea of T3, and the tuberculum of the rib was attached to the costal fovea of the transverse process of T3. For ribs positioned more caudal than the third rib, the costovertebral joints represent the same articulation system as that of the third rib. Namely, in giraffes, thoracic vertebrae typically possess ribs at the cranial and caudal joints of one vertebra. The T1 of giraffes has ribs only at the central area of the lateral side of the vertebral body without disturbing both joints of the vertebra.

In okapi, the costovertebral joint between the first rib and T1 displayed a structural similarity to the joint between the second rib and T2 in giraffes: the capitulum of the rib was attached at the cranial costal fovea of T1 without touching the caudal edge of the vertebral body of C7. In all ribs except the first, the capitulum of the rib was jointed to the socket formed by the caudal and cranial costal foveae of the two adjacent vertebrae, and the tuberculum of the rib articulated with the costal fovea of the transverse process. Thus, the thoracic vertebrae of okapi, including T1, generally possess the ribs on the cranial and caudal joints of the vertebral body.

### 3.3 Muscular structure

**Figure 3.**
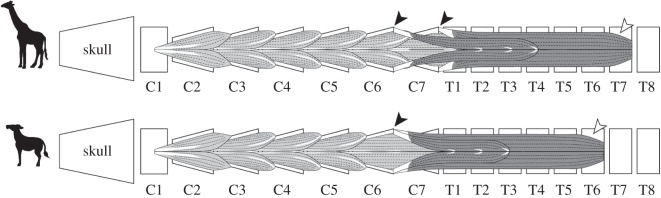
Schematic of muscular structure of *musculus longus colli* in the giraffe (upper) and okapi (lower). The diagrammatic illustration coloured by light grey indicates the structure of the cervical part of the *musculus longus colli*. The dark grey illustration shows the attachment area of the thoracic part of the muscle. The black arrowheads indicate the tendinous insertion of the thoracic part of the muscle, and the open arrowheads indicate the most caudal attachment area of the muscle.

In giraffes, the *musculus longus colli* comprised partly fused fascicles covering the ventral aspect of vertebral bodies of cervical and thoracic vertebrae from atlas to T7 and was divided into cervical and thoracic portions (figure 3). The cervical part arose on the concave ventral surfaces from C2 to T2 and was composed of six incompletely separated bundles. The five cranial bundles originated from the ventral edge of the ventral tubercles between C3 and C7, were directed craniomedially and partially terminated on the ventral spine of two preceding vertebrae by tendinous fibres. The most caudal and deepest bundle arose from the hypapophysial tubercles of T1 and T2, following a parallel course on the ventral surface between C7 and T2. The thoracic part comprised two fascicles that were partly fused. The medial part of the bundle arose from the hypapophysial tubercles between T2 and T4; it inserted on the caudal edge of the dorsal tubercle of C6 as a tendon. The lateral part of the bundle originated from the convex surfaces between T2 and T7 and terminated on the dorsal tubercle of C7 as a tendon. The muscle attached to the ventrolateral surface of the vertebral body of T1 without tendinous origin and insertion.

In okapi, the muscle arose on the ventral surface of the vertebral bodies from the atlas to T6; the cervical part attached on all cervical vertebrae and the first thoracic vertebra, and the thoracic part extended from the six most cranial thoracic vertebrae to C6 (figure 3). The components of the cervical part were five bundles; the four cranial bundles were located on the ventral surface from atlas to C5 and were directed craniomedially, and the most caudal bundle was distributed on the ventral area between C6 and T1 and ran parallel to the vertebral column. No obvious tendinous insertion was confirmed in C7. The thoracic part of the muscle arose on the ventral surfaces of the vertebral bodies from T1 to T6 and terminated on the caudal edge of the ventral lamella of C6 as two tendinous fasciculi. This muscle was located on the ventrolateral space of C7 without tendinous origin and insertion. A comparison of the muscular structure of the giraffe and okapi revealed that the attachment area of *musculus longus colli* in the giraffe was expanded posteriorly from T1 to T2 in the cervical part and that the origin and insertion of the muscle were partly shifted posteriorly in the thoracic part (figure 3).

## 4. Discussion

### 4.1 Behavioural advantages of the additional movable vertebra in the giraffe neck

A long neck has evolved independently in various taxa and consequently plays an important role in expanding the reachable space during foraging, improving predator detection and displaying dominance in competing males [21,29]. The long neck of the giraffe is the most famous example for understanding the evolution of neck elongation. Its neck has been considered to have evolved as a result of the adaptive evolution to high browsing behaviour and/or combat behaviour between males [30–32]. The height of the head would significantly influence their fitness. In giraffes, the maximum reachable height of the head is elevated by their long necks and limbs [33]. The forelimbs of giraffes are longer than the hind limbs; therefore, their trunk slopes from the cranial to the caudal direction, although the common ruminants, including okapi, show a horizontal trunk [9,34]. The pronounced slope of the trunk in the giraffe facilitates elevating the position of the cervicothoracic transition of the axial skeleton, resulting in a rise of the entire neck. The adaptive modifications of the body plan of the giraffe pose difficulties when lowering the head to the ground surface level for drinking water [33]. As a consequence, the development of a method of lowering the head and neck to facilitate the extension of the dorsoventral reachable space of the head was necessitated.

The flexibility of the axial skeleton is generally determined by two variables: the total number of movable vertebrae and the maximum mobility of each vertebral joint [35,36]. The mammalian thoracic region is rigid to facilitate respiration during locomotion and to counteract loading forces transmitted from the limbs [37,38]. Previous studies assumed that the majority of the head and neck movement occurred in the cervical region [16,21,39,40], suggesting that the total number of movable vertebrae is at a constant seven in the mammalian neck. Nevertheless, our study proved the expansion of the motion range in C7 of the giraffe and the mobility acquisition in T1 of the giraffe (figure 1*a*).

The head and neck of mammals are considered collectively as a loaded beam that is supported at one end only by attachment to the cranial end of the trunk region [11]. The cervical spine is regarded as an extended third-class lever: the resistance occurs on one side of an effort force, whereas the fulcrum is located on the other side [41]. Our findings suggest that the T1/T2 joint complex in giraffes acts as a fulcrum in this system. Conversely, the C7/T1 joint complex works as a fulcrum in okapi. It is predicted that a small increment of the dorsoventral mobility in the C7/T1 and T1/T2 joints in the giraffe would result in a large displacement at the cranial end level of the cervical spine, owing to their extraordinarily long neck.

### 4.2 Kinematic characteristics of C7 and T1 in the giraffe

The articulation of the rib is a principal factor restricting intervertebral flexion [38]. The rib typically articulates with the vertebrae through two positions in mammals: the costal fovea of the transverse process and the socket formed by the caudal and cranial costal foveae of two adjacent vertebrae [42]. This pattern was confirmed in the rib articulations in giraffe and okapi and imposes a fixed distance between the caudal costal fovea of a vertebra and the transverse fovea of the posterior adjacent vertebra, thereby imposing a strict limitation on vertebral flexion [38]. However, in the giraffe, the capitula of the first and second ribs did not make contact with the vertebral bodies of the adjacent vertebrae; consequently, in this region, the immobilization distance resulting from the rib articulation did not affect the vertebral flexion in the C7/T1 and T1/T2 joints, suggesting that the change of articular positions of the capitula of ribs in giraffes might minimize the restriction of vertebral mobility imposed by ribs around T1.

**Figure 4.**
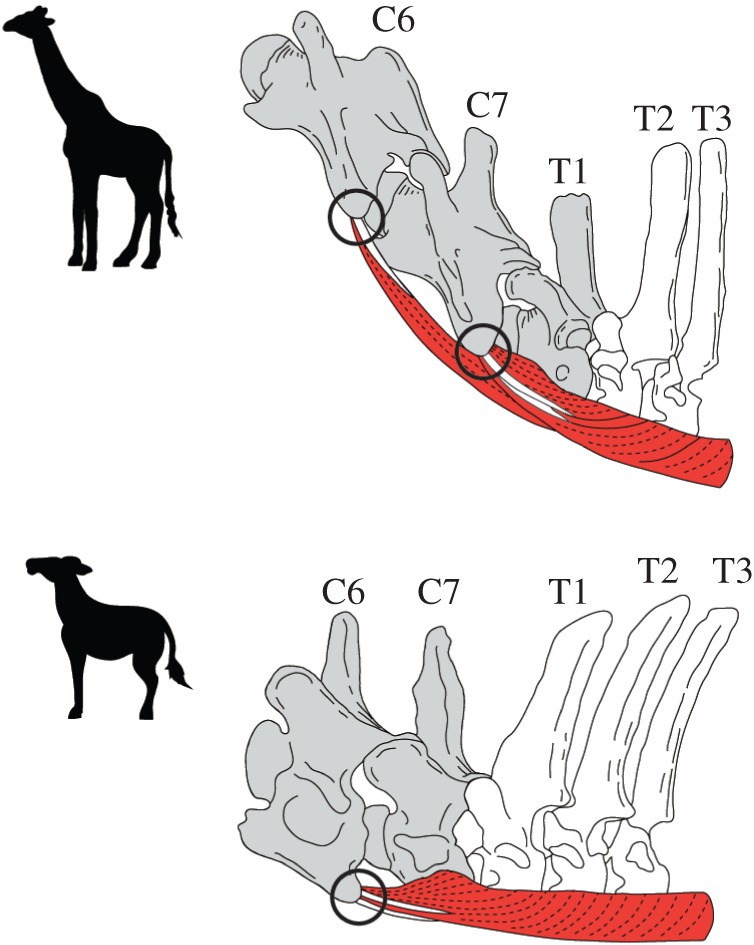
Functional musculoskeletal model of the neck movement at the cervicothoracic area in giraffes and okapi. The vertebrae coloured in grey possess the morphological characteristics closely related to high intervertebral flexibility and contain the insertion of the thoracic part of *musculus longus colli*. The white vertebrae display the characteristics representing low flexibility and have the origin of the muscle. The illustration of a muscle represents the attachment area of the thoracic part of *musculus longus colli* and demonstrates the muscular system generating the force to rotate the vertebrae at the cervicothoracic area. The circles indicate the points of the concentration of the force rotating the vertebra generated by this muscle.

The attachment area of *musculus longus colli* in giraffes demonstrates that the rotational force generated by the thoracic part of this muscle tends to be concentrated to their C6 and C7 by the strong tendons (figure 4). Although the force might act on T1, it would be lower than the power exerted in C6 and C7 because of the absence of the tendinous insertion in T1 of giraffes. Conversely, in okapi, the muscle would work more intensively on C6 than on C7, as C7 does not possess tendinous insertion (figure 4). The T1 of okapi does not possess the insertion; therefore, the muscle does not produce the rotational force on the vertebra. The vertebral movement inferred by the muscular structure is consistent with the result of our flexion experiment.

According to previous osteological studies, the C7 of giraffes possesses a long vertebral body, a round articular surface at the caudal joint between the vertebral bodies and large articular facets directed laterally on the posterior articular processes (table 2) [4–7]. These characteristics are generally observed in the vertebrae from C2 to C6 and could be regarded as the features representing high intervertebral flexibility, based on prior studies on the relationships between the vertebral shape and mobility [16,17,20,22,23]. The thoracic vertebrae from T2 to T14 display the vertebral shape representing low intervertebral mobility [16,17,20,22,23]. Namely, they present a short vertebral length, a flat articular surface at the caudal joint of the vertebral body, a long neural spine and small articular facets directed medially, just beneath the neural spine with no processes (table 2) [6,7]. Additionally, the T1 of giraffes represents the intermediate characteristics between the general cervical and thoracic vertebrae [6,7]. The vertebral body of T1 in giraffes is slightly longer than the other thoracic vertebrae and shows an intermediate length between the cervical and thoracic vertebrae [4]. T1 possesses two paired, large articular facets on the small posterior articular processes beneath the neural spine [7]. The vertebra presents a flat articular surface on the joint between T1 and T2. The long vertebral body, the large articular facets, and the posterior articular process would contribute to facilitating a higher vertebral mobility than that of general thoracic vertebrae [17,20,22,23]; however, the flat articular surface would enhance the rigidity between the joints [20]. This suggests that T1 of the giraffe potentially possesses a mobility that is intermediate of that between the general cervical and thoracic vertebrae.

**Table 2.** Morphological characteristics of a vertebra in relation to the mobility at cervicothoracic area in giraffes and okapi. (Dark grey area indicates the characteristics relating to the intervertebral stiffness, and the open area shows the characteristics representing the intervertebral flexibility. The light grey area is the intermediate characteristics between them. Morphological characteristics were described in previous studies [4–8]. Kinematic interpretation of the vertebral morphology is compliant with prior works [14–17,20,22,23].)

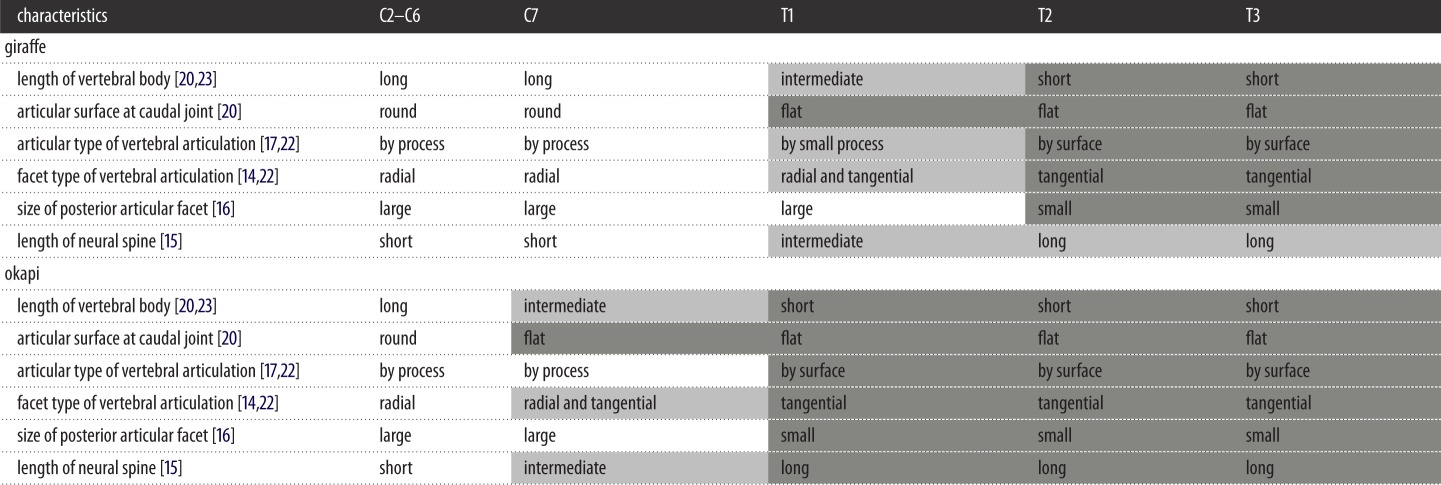

Conversely, the T1 of okapi possesses the same vertebral shape as the remaining thoracic vertebrae, representing low vertebral mobility [6]. In okapi, the intermediate shape is confirmed in C7 [6], suggesting that C7 of okapi presents lower mobility than the remaining cervical vertebrae. The vertebral morphology corresponds with our results regarding the expansion and acquisition of the vertebral flexibility in the C7/T1 and T1/T2 joints in the giraffe.

Solounias [6] hypothesized that the vertebra traditionally identified as T1 might be actually regarded as ‘the eighth cervical vertebra’ based on specific anatomical characteristics of the general cervical vertebrae; however, the first thoracic vertebra has been overlooked as merely one of thoracic vertebrae as it possesses the movable ribs. Our study provided anatomical evidence that T1 of the giraffe played a kinematic role corresponding to the C7 of general ruminants even if the vertebra articulated with the sternum via the movable ribs. Note that, in this study, there is no evidence supporting that T1 is originally cervical vertebra. Our results indicate that the morphological shift of the vertebrae of giraffes at the cervicothoracic area has great significance in understanding evolutionary functional plasticity of the mammalian vertebral column rather than in discussing the number of cervical vertebrae.

### 4.3 Developmental background of the morphological shift of the vertebral column

The musculoskeletal structure of the axial skeleton consists of two elements derived from different developmental backgrounds: primaxial and abaxial elements [43–46]. The vertebrae, vertebral ribs and epaxial and hypaxial muscles develop only from somitic mesoderm and are classified as the primaxial elements. The sternum and limbs originate from lateral plate mesoderm and are identified as the abaxial elements [43]. The abaxial domain contains somitic cells that migrate into the lateral plate environment early during development and mix with lateral plate cells [43]. Curiously, in mice, the first rib is completely identified as an abaxial element [46]. The expression patterns of *Hox* genes, regulating the morphology of the vertebral column and rib cage [45,47–51], are independent between the primaxial and abaxial domains [43,50,52]. In the abaxial domain, the migrating somitic cells adopt the *Hox* expression of the lateral plate mesoderm into which they migrate [53]. Additionally, a primaxial patterning possesses a colinearity of the phenotype, whereas an abaxial patterning is not colinear [50]. Colinearity is the phenomenon that the order of the *Hox* genes along the chromosome corresponds with the order of the expression domains of the genes along the anterior–posterior axis of the embryo. Mutations of the *Hox* genes cause the homeotic transformation in primaxial elements, whereas it results in serious patterning defects in the abaxial elements [50,54–56].

In the light of the above evidence obtained from previous genetic studies, we considered the possibility that the mutations of *Hox*5 and/or *Hox*6 specific to the primaxial domain would have resulted in the transformations of the musculoskeletal structure in the neck of giraffes. Both genes are important for the specification of the identity of the vertebral column between C6 and T1 [57]. *Hox*6 genes function to induce ribs by regulating *Myf*5/*Myf*6 expression, suggesting that their expression pattern is responsible primarily for the regionalization of the vertebral column between the cervical and thoracic vertebrae [51]. When we consider the genetic background of the development of the vertebrae and ribs in giraffes along with the evidence obtained from mice, it could be assumed that the mutations of the *Hox*5 and/or *Hox*6 genes specific to the primaxial domain caused the morphological transformations of the vertebrae and ribs, with the exception of the first rib. From the study of mice, it has been predicted that the development of the first rib would be regulated by the *Hox* expression pattern in the abaxial domain [46], indicating that the phenotype of the first rib is unaffected by the mutations of the *Hox* genes specific to the primaxial domain. Therefore, it could now be explained that the mutations of the *Hox* genes specific to the primaxial domain causes the morphological transformations of the vertebrae in giraffes without displacing the position of the rib cage.

## 5. Conclusion

We quantitatively demonstrated the expansions of the motion range in C7 and T1 of giraffes by the flexion experiment. The muscular system generating a vertebral rotation was partly modified around the cervicothoracic region, which supported the result of the estimation of the vertebral mobility. The modifications of the attachment position of the capitula of the first and second ribs in giraffes minimize the restriction on vertebral flexion imposed by ribs, thereby enabling notable flexion of the joint between T1 and T2. The addition of movable articulation to the thoracic region results in a large displacement of the head, even if the mobility is relatively smaller than that of the cervical joints. Based on our findings, we assert the evolutionary importance of the increased mobility at the base of the neck in terms of the advanced adaptation to high browsing in giraffes. Under the rigorous cervical constraint, giraffes have achieved the functional novelties at the cervicothoracic region by subtle modifications in their existing musculoskeletal system.

Recent studies have reconsidered the regionalization of the vertebral column by focusing not on the presence of movable ribs but on the morphological characteristics of the vertebrae [58–60]. Tree sloths are well known to exhibit an abnormal number of cervical vertebrae: *Bradypus* (three-toed sloths) possess eight to ten cervical vertebrae, whereas *Choloepus* (two-toed sloths) possess five to eight cervical vertebrae [58]. However, the morphological characteristics and the ossification pattern of the vertebrae have demonstrated that the boundary between the cervical and thoracic was identified between seventh and eighth vertebrae, as is the case in other mammals [58,59,61]. These studies regarded the abnormal number of cervical vertebrae in sloths as rib-less thoracic vertebrae in the neck or rib-bearing cervical vertebrae in the thorax [59]. They proposed the idea that the constraint of the cervical vertebrae was actually conserved even in the sloths that had historically been the most famous group thought to deviate from the constraint [58]. This idea offered new insight, clearly different from the previous perception, into the regionalization of the vertebral column in mammals. Our findings additionally provide evidence that the vertebral regionalization in giraffes is slightly shifted posteriorly at the cervicothoracic transitional region without changing the traditional border, based on the presence of movable ribs. This suggests the possibility that the vertebral regionalization is additionally diversified in other mammals that maintain the number of cervical vertebrae. We advocate that subtle mutations of the *Hox* genes can induce the striking structural and functional novelties in the conservative existing system.

The flexibility of the neck is of major importance in inferring the neck posture and the feeding strategy in extinct long-necked species, including sauropods [29,62]. Many studies have evaluated the flexibility of the sauropod neck in the light of vertebral morphology [16,19,23,62–66], whereas they have not paid any attention to thoracic vertebrae. However, in diplodocid sauropods, it was reported that the morphological characteristics representing high intervertebral flexibility were found in three to four most anterior thoracic vertebrae [67]. This indicates the possibility that diplodocid sauropods possess movable thoracic vertebrae. The dorsoventral movement of the thoracic vertebra effectively enlarges the displacement at the level of the head. The estimated reachable area of the head will strongly depend on whether we consider the thoracic vertebrae. The evidence that the giraffe gained a movable joint in the thoracic region emphasizes the need for evaluating the flexibility of the thoracic vertebrae. Our discovery provides new insight into the evolutionary process leading to the development of the neck of the giraffe and into the reconstruction of neck function and posture in extinct long-necked animals.

## Data accessibility

The datasets supporting this article have been uploaded as the electronic supplementary material. The movie of the flexion experiment in a giraffe is uploaded as the electronic supplementary material, video S1. The three-dimensional models constructed from computed tomography images are provided as the electronic supplementary material, videos S2–S5.

## References

[1] Crompton AW, Jenkins FA 1973 Mammals from reptiles: a review of mammalian origins. *Ann. Rev. Earth Planet Sci.* 1, 131–155. (doi:10.1146/annurev.ea.01.050173.001023)

[2] Galis F 1999 Why do almost all mammals have seven cervical vertebrae? Developmental constraints, *Hox* genes and cancer. *J. Exp. Zool.* 285, 19–26. (doi:10.1002/(SICI)1097-010X(19990415)285:1<19::AID-JEZ3>3.0.CO;2-Z) 10327647

[3] Narita Y, Kuratani S 2005 Evolution of the vertebral formulae in mammals: a perspective on developmental constraints. *J. Exp. Zool.* 304, 91–106. (doi:10.1002/jez.b.21029) 10.1002/jez.b.21029 15660398

[4] Badlangana NL, Adams JW, Manger PR 2009 The giraffe (*Giraffa camelopardalis*) cervical vertebral column: a heuristic example in understanding evolutionary processes? *Zool. J. Linn. Soc.* 155, 736–757. (doi:10.1111/j.1096-3642.2008.00458.x)

[5] van Sittert SJ, Skinner JD, Mitchell G 2010 From fetus to adult: an allometric analysis of the giraffe vertebral column. *J. Exp. Zool.* 314B, 469–479. (doi:10.1002/jez.b.21353) 10.1002/jez.b.21353 20700891

[6] Solounias N 1999 The remarkable anatomy of the giraffe’s neck. *J. Zool.* 247, 257–268. (doi:10.1111/j.1469-7998.1999.tb00989.x)

[7] Lankester R 1908 On certain points in the structure of the cervical vertebrae of the okapi and the giraffe. *Proc. Zool. Soc. Lond.* 1908, 320–334. (doi:10.1111/j.1096-3642.1908.tb01845.x)

[8] Danowitz M, Solounias N 2015 The cervical osteology of *Okapia johnstoni* and *Giraffa camelopardalis*. *PLoS ONE* 10, e0136552 (doi:10.1371/journal.pone.0136552) 26302156 10.1371/journal.pone.0136552 PMC4547811

[9] Mitchell G, Skinner JD 2003 On the origin, evolution and phylogeny of giraffes *Giraffa camelopardalis*. *Trans. R. Soc. S. Afr.* 58, 51–73. (doi:10.1080/00359190309519935)

[10] Endo H, Koyabu D, Hayashida A, Oishi M, Kawada S, Komiya T 2009 The brachial plexus adapted to the semi-elongated neck in okapi. *Mamm. Study* 34, 209–212. (doi:10.3106/041.034.0405)

[11] Slijper EJ 1946 Comparative biologic-anatomical investigations on the vertebral column and spinal musculature of mammals. *Verh. Kon. Neder. Akad. Wetensch.* 42, 1–128.

[12] Townsend HGG, Leach DH, Fretz PB 1983 Kinematics of the equine thoracolumbar spine. *Equine Vet. J.* 15, 117–122. (doi:10.1111/j.2042-3306.1983.tb01732.x) 6873044 10.1111/j.2042-3306.1983.tb01732.x

[13] Townsend HGG, Leach DH 1984 Relationship between intervertebral joint morphology and mobility in the equine thoracolumbar spine. *Equine Vet. J.* 16, 461–465. (doi:10.1111/j.2042-3306.1984.tb01981.x) 6489309 10.1111/j.2042-3306.1984.tb01981.x

[14] Milne N 1991 The role of zygapophysial joint orientation and uncinate processes in controlling motion in the cervical spine. *J. Anat.* 178, 189–201. 1810926 PMC1260546

[15] Long JH Jr, Pabst DA, Shepherd WR, McLellan WA 1997 Locomotor design of dolphin vertebral columns: bending mechanics and morphology of *Delphinus delphis*. *J. Exp. Biol.* 200, 65–81. 9023994 10.1242/jeb.200.1.65

[16] Stevens KA, Parrish JM 1999 Neck posture and feeding habits of two Jurassic sauropod dinosaurs. *Science* 284, 798–800. (doi:10.1126/science.284.5415.798) 10221910 10.1126/science.284.5415.798

[17] Boszczyk BM, Boszczyk AA, Putz R 2001 Comparative and functional anatomy of the mammalian lumbar spine. *Anat. Rec.* 264, 157–168. (doi:10.1002/ar.1156) 11590593 10.1002/ar.1156

[18] Buchholtz EA 2001 Vertebral osteology and swimming style in living and fossil whales (Order: Cetacea). *J. Zool.* 253, 383–401. (doi:10.1017/S0952836901000164)

[19] Stevens KA 2002 DinoMorph: parametric modeling of skeletal structures. *Senck. leth.* 82, 23–34. (doi:10.1007/BF03043770)

[20] Buchholtz EA, Schur SA 2004 Vertebral osteology in Delphinidae (Cetacea). *Zool. J. Linn. Soc.* 140, 383–401. (doi:10.1111/j.1096-3642.2003.00105.x)

[21] Taylor MP, Wedel MJ, Naish D 2009 Head and neck posture in sauropod dinosaurs inferred from extant animals. *Acta Palaeontol. Pol.* 54, 213–220. (doi:10.4202/app.2009.0007)

[22] Kuznetsov AN, Tereschenko VS 2010 A method for estimation of lateral and vertical mobility of platycoelous vertebrae of tetrapods. *Paleontol. J.* 44, 209–225. (doi:10.1134/S0031030110020139)

[23] Stevens KA 2013 The articulation of sauropod necks: methodology and mythology. *PLoS ONE* 8, e78572 (doi:10.1371/journal.pone.0078572) 24205266 10.1371/journal.pone.0078572 PMC3812995

[24] Granatosky MC, Lemelin P, Chester SGB, Pampush JD, Schmitt D 2014 Functional and evolutionary aspects of axial stability in Euarchontans and other mammals. *J. Morph.* 275, 313–327. (doi:10.1002/jmor.20216). 24288155 10.1002/jmor.20216

[25] Graf W, de Waele C, Vidal PP 1995 Functional anatomy of the head-neck movement system of quadrupedal and bipedal mammals. *J. Anat.* 186, 55–74. 7649818 PMC1167272

[26] Smuts MM, Bezuidenhout AJ 1987 The muscular system. In *Anatomy of the dromedary*, pp. 59–104. Oxford, UK: Clarendon Press.

[27] Owen R 1839 Notes on the anatomy of the Nubian giraffe. *Trans. Zool. Soc. Lond.* 2, 217–243. (doi:10.1111/j.1469-7998.1839.tb00021.x)

[28] Murie J 1872 On the horns, viscera, and muscles of the giraffe; with a record of the post mortem examination of two specimens killed by a fire. *Ann. Mag. Nat. Hist.* 51, 177–195. (doi:10.1080/00222937208696563)

[29] Wilkinson DM, Ruxton GD 2012 Understanding selection for long necks in different taxa. *Biol. Rev.* 87, 616–630. (doi:10.1111/j.1469-185X.2011.00212.x) 22171805 10.1111/j.1469-185X.2011.00212.x

[30] Simmons RE, Scheepers L 1996 Winning by a neck: sexual selection in the evolution of giraffe. *Am. Nat.* 148, 771–786. (doi:10.1086/285955)

[31] Cameron EZ, du Toit JT 2007 Winning by a neck: tall giraffes avoid competing with shorter browsers. *Am. Nat.* 169, 130–135. (doi:10.1086/509940) 17206591 10.1086/509940

[32] Simmons RE, Altwegg R 2010 Necks-for-sex or competing browsers? A critique of ideas on the evolution of giraffe. *J. Zool.* 282, 6–12. (doi:10.1111/j.1469-7998.2010.00711.x)

[33] Pincher C 1949 Evolution of the giraffe. *Nature* 164, 29–30. (doi:10.1038/164029b0) 18153292 10.1038/164029b0

[34] Colbert EH 1938 The relationships of the okapi. *J. Mammal.* 19, 47–64. (doi:10.2307/1374281)

[35] Brainerd EL, Patek SN 1998 Vertebral column morphology, C-start curvature, and the evolution of mechanical defenses in tetraodontiform fishes. *Copeia* 1998, 971–984. (doi:10.2307/1447344)

[36] Preuschoft H, Klein N 2013 Torsion and bending in the neck and tail of sauropod dinosaurs and the function of cervical ribs: insights from functional morphology and biomechanics. *PLoS ONE* 8, e78574 (doi:10.1371/journal.pone.0078574) 24205268 10.1371/journal.pone.0078574 PMC3812989

[37] Schilling N, Hackert R 2006 Sagittal spine movements of small therian mammals during asymmetrical gaits. *J. Exp. Biol.* 209, 3925–3939. (doi:10.1242/jeb.02400) 16985208 10.1242/jeb.02400

[38] Filler AG 2007 Costal and articular constraints on axial motion. In *Axial character seriation in mammals: an historical and morphological exploration of the origin, development, use, and current collapse of the homology paradigm*, pp. 200–208. Boca Raton, FL: BrownWalker Press.

[39] Jeffcott LB 1979 Back problems in the horse: a look at past, present and future progress. *Equine Vet. J.* 11, 129–136. (doi:10.1111/j.2042-3306.1979.tb01324.x) 158523 10.1111/j.2042-3306.1979.tb01324.x

[40] Selbie WS, Thomson DB, Richmond FJR 1993 Sagittal-plane mobility of the cat cervical spine. *J. Biomech.* 26, 917–927. (doi:10.1016/0021-9290(93)90054-I) 8349717 10.1016/0021-9290(93)90054-i

[41] Davidovits P 2007 Static forces. In *Physics in biology and medicine*, 3rd edn, pp. 1–21. San Diego, CA: Academic Press.

[42] Liebich HG, König HE 2006 Axial skeleton (sleleton axiale). In *Veterinary anatomy of domestic mammals: textbook and colour atlas* (eds HE König, HG Liebich), pp. 49–112. Stuttgart, Germany: Schattauer Verlag.

[43] Burke AC, Nowicki JL 2003 A new view of patterning domains in the vertebrate mesoderm. *Dev. Cell* 4, 159–165. (doi:10.1016/S1534-5807(03)00033-9) 12586060 10.1016/s1534-5807(03)00033-9

[44] Nowicki JL, Takimoto R, Burke AC 2003 The lateral somitic frontier: dorso-ventral aspects of anterio-posterior regionalization in avian embryos. *Mech. Dev.* 120, 227–240. (doi:10.1016/S0925-4773(02)00415-X) 12559495 10.1016/s0925-4773(02)00415-x

[45] Wellik DM 2007 *Hox* patterning of the vertebrate axial. *Dev. Dyn.* 236, 2454–2463. (doi:10.1002/dvdy.21286) 17685480 10.1002/dvdy.21286

[46] Durland JL, Sferlazzo M, Logan M, Burke AC 2008 Visualizing the lateral somitic frontier in the Prx1Cre transgenic mouse. *J. Anat.* 212, 590–602. (doi:10.1111/j.1469-7580.2008.00879.x.) 18430087 10.1111/j.1469-7580.2008.00879.x PMC2409079

[47] Burke AC, Nelson CE, Morgan BA, Tabin C 1995 *Hox* genes and the evolution of vertebrate axial morphology. *Development* 121, 333–346. 7768176 10.1242/dev.121.2.333

[48] Johnson DR, O’Higgins P 1996 Is there a link between changes in the vertebral ‘*hox code*’ and the shape of vertebrae? A quantitative study of shape change in cervical vertebral column of mice. *J. Theor. Biol.* 183, 89–93. (doi:10.1006/jtbi.1996.0204) 8959111 10.1006/jtbi.1996.0204

[49] Wellik DM, Capecchi MR 2003 *Hox*10 and *Hox*11 genes are required to globally pattern the mammalian skeleton. *Science* 301, 363–367. (doi:10.1126/science.1085672) 12869760 10.1126/science.1085672

[50] McIntyre DC, Rakshit S, Yallowitz AR, Loken L, Jeannotte L, Capecchi MR, Wellik DM 2007 *Hox* patterning of the vertebrate rib cage. *Development* 134, 2981–2989. (doi:10.1242/dev.007567) 17626057 10.1242/dev.007567

[51] Vinagre T, Moncaut N, Carapuço M, Nóvoa A, Bom J, Mallo M 2010 Evidence for a myotomal *Hox*/*Myf* cascade governing nonautonomous control of rib specification within global vertebral domains. *Dev. Cell* 18, 655–661. (doi:10.1016/j.devcel.2010.02.011) 20412779 10.1016/j.devcel.2010.02.011

[52] Nowicki JL, Burke AC 2000 *Hox* genes and morphological identity: axial versus lateral patterning in the vertebrate mesoderm. *Development* 127, 4265–4275. 10976057 10.1242/dev.127.19.4265

[53] Burke AC, Nowicki JL 2001 *Hox* genes and axial specification in vertebrates. *Am. Zool.* 41, 687–697. (doi:10.1093/icb/41.3.687)

[54] Jeannotte L, Lemieux M, Charron J, Poirier F, Robertson EJ 1993 Specification of axial identity in the mouse: role of the *Hox*a-5 (*Hox*1.3) gene. *Genes Dev.* 7, 2085–2096. (doi:10.1101/gad.7.11.2085) 7901120 10.1101/gad.7.11.2085

[55] Kostic D, Capecchi MR 1994 Targeted disruptions of the murine *Hoxa*-4 and *Hoxa*-6 genes result in homeotic transformations of components of the vertebral column. *Mech. Dev.* 46, 231–247. (doi:10.1016/0925-4773(94)90073-6) 7918106 10.1016/0925-4773(94)90073-6

[56] Rancourt DE, Tsuzuki T, Capecchi MR 1995 Genetic interaction between *hoxb*-5 and *hoxb*-6 is revealed by nonallelic noncomplementation. *Genes Dev.* 9, 108–122. (doi:10.1101/gad.9.1.108) 7828847 10.1101/gad.9.1.108

[57] Medina-Martínez O, Bradley A, Ramírez-Solis R 2000 A large targeted deletion of *Hoxb1-Hoxb9* produces a series of single-segment anterior homeotic transformations. *Dev. Biol.* 222, 71–83. (doi:10.1006/dbio.2000.9683) 10885747 10.1006/dbio.2000.9683

[58] Buchholtz EA, Stepien CC 2009 Anatomical transformation in mammals: developmental origin of aberrant cervical anatomy in tree sloths. *Evol. Dev.* 11, 69–79. (doi:10.1111/j.1525-142X.2008.00303.x) 19196334 10.1111/j.1525-142X.2008.00303.x

[59] Hautier L, Weisbecker V, Sánchez-Villagra MR, Goswami A, Asher RJ 2010 Skeletal development in sloths and the evolution of mammalian vertebral patterning. *Proc. Natl Acad. Sci. USA* 107, 18 903–18 908. (doi:10.1073/pnas.1010335107) 10.1073/pnas.1010335107 PMC2973901 20956304

[60] Head JJ, Polly PD 2015 Evolution of the snake body form reveals homoplasy in amniote *Hox* gene function. *Nature* 520, 86–89. (doi:10.1038/nature14042) 25539083 10.1038/nature14042

[61] Bell T 1833 Observations on the neck of the three-toed sloths, *Bradypus tridactylus*, Linn. *Trans. Zool. Soc. Lond.* 1, 113–116. (doi:10.1111/j.1096-3642.1835.tb00608.x)

[62] Stevens KP, Parrish JM 2005 Digital reconstructions of sauropod dinosaurs and implications for feeding. In *The sauropods: evolution and paleobiology* (eds KC Rogers, JA Vilson), pp. 178–200. Berkeley, CA: University of California Press.

[63] Dzemski G, Christian A 2007 Flexibility along the neck of the ostrich (*Struthio camelus*) and consequences for the reconstruction of dinosaurs with extreme neck length. *J. Morph.* 268, 701–714. (doi:10.1002/jmor.10542) 17514722 10.1002/jmor.10542

[64] Taylor MP, Wedel MJ 2013 The effect of intervertebral cartilage on neural posture and range of motion in the necks of sauropod dinosaurs. *PLoS ONE* 8, e78214 (doi:10.1371/journal.pone.0078214) 24205163 10.1371/journal.pone.0078214 PMC3812996

[65] Christian A 2002 Neck posture and overall body design in sauropods. *Fossil Rec.* 5, 271–281. (doi:10.1002/mmng.20020050116)

[66] Taylor MP 2014 Quantifying the effect of intervertebral cartilage on neutral posture in the necks of sauropod dinosaurs. *PeerJ* 2, e712 (doi:10.7717/peerj.712/) 25551027 10.7717/peerj.712 PMC4277489

[67] Upchurch P 2000 Neck posture of sauropod dinosaurs. *Science* 287, 547 (doi:10.1126/science.287.5453.547b)

